# Human equilibrative nucleoside transporter 1 (hENT1) expression as a predictive biomarker for gemcitabine chemotherapy in biliary tract cancer

**DOI:** 10.1371/journal.pone.0209104

**Published:** 2018-12-17

**Authors:** Jaihwan Kim, Haeryoung Kim, Jong-chan Lee, Jin Won Kim, Woo Hyun Paik, Sang Hyub Lee, Jin-Hyeok Hwang, Ji Kon Ryu, Yong-Tae Kim

**Affiliations:** 1 Department of Internal Medicine, Seoul National University Bundang Hospital, Seoul National University College of Medicine, Seongnam, Korea; 2 Department of Pathology, Seoul National University Hospital, Seoul National University College of Medicine, Seoul, Korea; 3 Department of Internal Medicine and Liver Research Institute, Seoul National University Hospital, Seoul National University College of Medicine, Seoul, Korea; University of South Alabama Mitchell Cancer Institute, UNITED STATES

## Abstract

Gemcitabine is a principal chemotherapeutic agent for biliary tract cancer (BTC). Expression of human equilibrative nucleoside transporter 1 (hENT1) is regarded as a potential predictive biomarker for a gemcitabine response in some cancers. This study was conducted to investigate the association between hENT1 expression and the effects of gemcitabine on BTC cell lines and on patients with advanced BTC receiving gemcitabine-based chemotherapy. A total of four BTC cell lines, HuCCT1, SNU-478, SNU-1079, and SNU-1196, were tested. mRNA and protein expression levels of hENT1 were measured by quantitative reverse-transcription polymerase chain reaction and western blotting, respectively. Cell viability after gemcitabine treatment was measured in a chemosensitivity assay. For clinical assessment, 40 patients with unresectable or recurrent BTC who were treated with gemcitabine (1000 mg/m^2^) and cisplatin (25 mg/m^2^) between June 2012 and May 2014 were enrolled. Among the four cell lines, SNU1196 showed the highest mRNA and protein levels of hENT1. Expression of hENT1 showed a linear correlation with the log value of the half-maximal inhibitory concentration of gemcitabine. During incubation with gemcitabine, pretreatment with hENT1-specific small interfering RNA (siRNA) resulted in higher cell viability than that in samples pretreated with control siRNA. In a clinical evaluation, the median progression-free survival was 24 and 11 weeks among patients with strong and weak intratumoral hENT1 immunohistochemical staining (P = 0.05), and the median overall survival was 52 and 26 weeks (P = 0.15), respectively. In conclusion, this study showed that increased hENT1 expression is associated with a stronger toxic effect of gemcitabine on BTC cell lines. The clinical outcomes in this study suggest that increased intratumoral hENT1 immunohistochemical staining is a possible biomarker predicting better therapeutic effects of gemcitabine on patients with advanced BTC. Further studies are needed to determine the precise role of hENT1 in BTC.

## Introduction

Biliary tract cancer (BTC) is a relatively uncommon malignant tumor that originates in the biliary tract including the intra- and extrahepatic bile duct and gallbladder. This cancer has a poor prognosis because of the advanced stage at diagnosis in many cases or frequent recurrence even after curative resection [1, 2]. Thus, most patients with unresectable or recurrent BTC depend on palliative systemic chemotherapy. A gemcitabine plus cisplatin (GP) regimen was recently accepted as first-line chemotherapy [3]. Nonetheless, the efficacy of GP varies among individuals. Identifying a useful predictive biomarker for a GP response in BTC is necessary because of the cost and toxicity associated with this treatment.

Gemcitabine is an analog of cytidine and a commonly used chemotherapeutic agent for various cancers, including BTC. Just as other nucleoside analogs, it is a prodrug that requires cellular uptake and intracellular phosphorylation. Human equilibrative nucleoside transporter 1 (hENT1) and human concentrative nucleoside transporters 1 and 3 (hCNT1 and -3) are important for the transport of gemcitabine into the cell. Inside the cell, gemcitabine is metabolized to active gemcitabine diphosphate and triphosphate by deoxycytidine kinase [4, 5]. During this process, active metabolites inhibit ribonucleotide reductase subunits 1 and 2, whose expression is associated with gemcitabine resistance [6]. Gemcitabine is mainly inactivated by cytidine deaminase [7].

Recently, intratumoral hENT1 has been reported as a candidate predictive biomarker for gemcitabine therapy responses in various cancers [8–11]. Nevertheless, its predictive value in BTC, particularly in patients at an unresectable stage, is unclear [12–14].

The present study was conducted to investigate the association between hENT1 expression and the effects of gemcitabine both on BTC cell lines and on patients with advanced BTC who follow a GP regimen.

## Materials and methods

### 1. *In vitro* experiments

#### BTC cell lines and chemicals

Four BTC cell lines were analyzed in this study. The HuCCT1 cell line (intrahepatic cholangiocarcinoma origin) was purchased from the RIKEN BioResource Center (Ibaraki, Japan). The SNU-478 (ampulla of Vater adenocarcinoma origin), SNU-1079 (intrahepatic cholangiocarcinoma origin), and SNU-1196 (extrahepatic cholangiocarcinoma origin) cell lines were acquired from the Korean Cell Line Bank (Seoul, Korea). All the cell lines were cultured in the RPMI 1640 medium (Gibco, Grand Island, NY, USA) supplemented with 10% of fetal bovine serum (Gibco) and 1% of a penicillin/streptomycin solution (Gibco) and were maintained at 37°C and 5% atmospheric CO_2_. Gemcitabine was provided by Lilly Korea (Seoul, Korea) for *in vitro* experiments. The company was not involved in anything related to the study.

Total-RNA isolation, cDNA synthesis, and quantitative reverse-transcription polymerase chain reaction (RT-PCR)

Total-RNA was isolated from the cells using the RNeasy Plus Mini Kit (Qiagen, Hilden, Germany), and cDNA was synthesized with the High Capacity cDNA Reverse Transcription Kit (Applied Biosystems, Foster City, CA, USA). The primer sets for RT-PCR analysis of *hENT1* and *GAPDH* were as follows: *hENT1*, 5′-CAGGCAAAGAGGAATCTGGA-3′ and 5′-GGCCCAACCAGTCAAAGATA-3′; *GAPDH*, 5′-TTCACCACCATGGAGAAGGC-3′ and 5′-GGCATGGACTGTGGTCATGA-3′. The primers were designed by means of published sequence data from GenBank (https://www.ncbi.nlm.nih.gov/genbank/) and a BLAST search (https://blast.ncbi.nlm.nih.gov/Blast.cgi/). The Power SYBR Green PCR Master Mix (Applied Biosystems) served as the reaction mixture, in a 20 μL volume in each reaction capillary. The mRNA expression of the gene under study was normalized to the corresponding expression of *GAPDH*.

#### Western blot analysis

Cells were washed rapidly with ice-cold PBS and lysed in RIPA buffer (Cell Signaling Technology, Danvers, MA, USA) containing 1 mM of the protease inhibitor PMSF (Cell Signaling Technology) and the Protease Inhibitor Cocktail (Sigma-Aldrich, St. Louis, MO, USA). The cell extracts were centrifuged at 13,000 rpm for 20 min. The total-protein concentration in the supernatant was measured with the BCA reagent (Pierce, Rockford, IL, USA). For each sample, equal amounts of total protein were denatured and separated by sodium dodecyl sulfate 10% polyacrylamide gel electrophoresis and then transferred onto a nitrocellulose membrane. The membranes were blocked for 1 h in 5% skimmed milk powder solution composed of Tris-buffered saline (TBS) containing 0.1% Tween (TBST). Membranes were incubated overnight at 4°C with primary antibodies against hENT1 (Abcam, Cambridge, UK) and β-actin (Cell Signaling Technology). After three washes with TBST, the membranes were incubated with the appropriate horseradish peroxidase–conjugated secondary antibodies for 2 h (Cell Signaling Technology). Bound antibodies were detected by means of the ECL reagent (Amersham Pharmacia Biotech, Buckinghamshire, UK) and an X-ray film (AGFA, Mortsel, Belgium).

#### RNA interference and a cell viability assay

Cells were cultivated in culture plates to 60–70% confluence. After a wash with PBS, the cells were treated with the Lipofectamine RNAiMAX reagent (Invitrogen, Carlsbad, CA, USA) mixed with hENT1-targeting small interfering RNA (siRNA; Assay ID s4694; Ambion, Foster City, CA, US) or nontargeting control siRNA (catalog No. AM4620; Ambion) at a final amount of 10 nmol in Opti-MEM (Gibco). At 72 h after siRNA treatment, cells were harvested to examine silencing efficiency and the effect of the hENT1 knockdown.

These hENT1 knockdown cells and control cells were seeded at 3,000–5,000 per well in 96-well plates. After overnight incubation, the cells were treated with gemcitabine (0–100 μM) for 72 h. Cell viability was determined using the Cell Titer-Glo Luminescent Cell Viability Assay Kit (Promega, Madison, WI, USA). Luminescence was measured on a LMAXII 384 microplate reader (Molecular Devices, Sunnyvale, CA, USA). Half-maximal inhibitory concentrations (IC_50_) were calculated in the SigmaPlot software.

### 2. Clinical validation

#### Patients

Between June 2012 and May 2014, 193 patients with BTC who received gemcitabine-based therapy were identified via electronic medical records at Seoul National University Bundang Hospital. Among them, 153 patients were excluded due to the following: gemcitabine as adjuvant therapy, single gemcitabine or combination with other agents as palliative therapy, a dose different from that in the current first-line regimen (gemcitabine 1,000 mg/m^2^ and cisplatin 25 mg/m^2^) [3], or a lack of formalin-fixed paraffin-embedded block samples for immunohistochemistry. Eventually, 40 patients were enrolled, and their medical records were retrospectively reviewed. Details regarding enrolled patients in this study are provided in S1 Table. The study protocol was approved by the Institutional Review Board of Seoul National University Bundang Hospital (IRB No.: B-1804-465-101). Informed consent was waived by the board.

#### hENT1 immunohistochemistry

Immunohistochemical analysis of hENT1 was performed on 4-μm-thick unstained sections of formalin-fixed paraffin-embedded BTC specimens on an automated Ventana BenchMark XT system with an anti-hENT1 SP120 rabbit monoclonal antibody (Ventana Medical Systems, Inc., Tucson, AZ, USA) as previously reported [15].

Grading of hENT1 staining was performed by an experienced pancreaticobiliary pathologist (HK) as follows: 2+, membranous staining in more than 50% of tumor cells; 1+, membranous staining in 5–50% of tumor cells; 0, no hENT1 staining or staining in <5% of tumor cells. Grade 0 or 1+ was regarded as low hENT1 expression, and grade 2+ was considered high hENT1 expression according to another study [15].

### 3. Statistics

The *in vitro* experimental results shown are representative of three or more independent experiments. Pearson’s χ^2^ test or Fisher’s exact test was conducted to determine the differences between categorical variables. Continuous variables were compared by the Mann–Whitney *U* test. Kaplan–Meier analysis was carried out to generate survival curves and calculate the median survival periods, which were compared by the log rank test. A two-sided P value less than 0.05 indicated statistical significance. All statistical analyses were performed in GraphPad Prism 7.0 (GraphPad Software, La Jolla, CA, USA) and in R software v3.4.3 (The R Development Core Team).

## Results

### 1. Baseline mRNA and protein expression of hENT1

**Fig 1:**
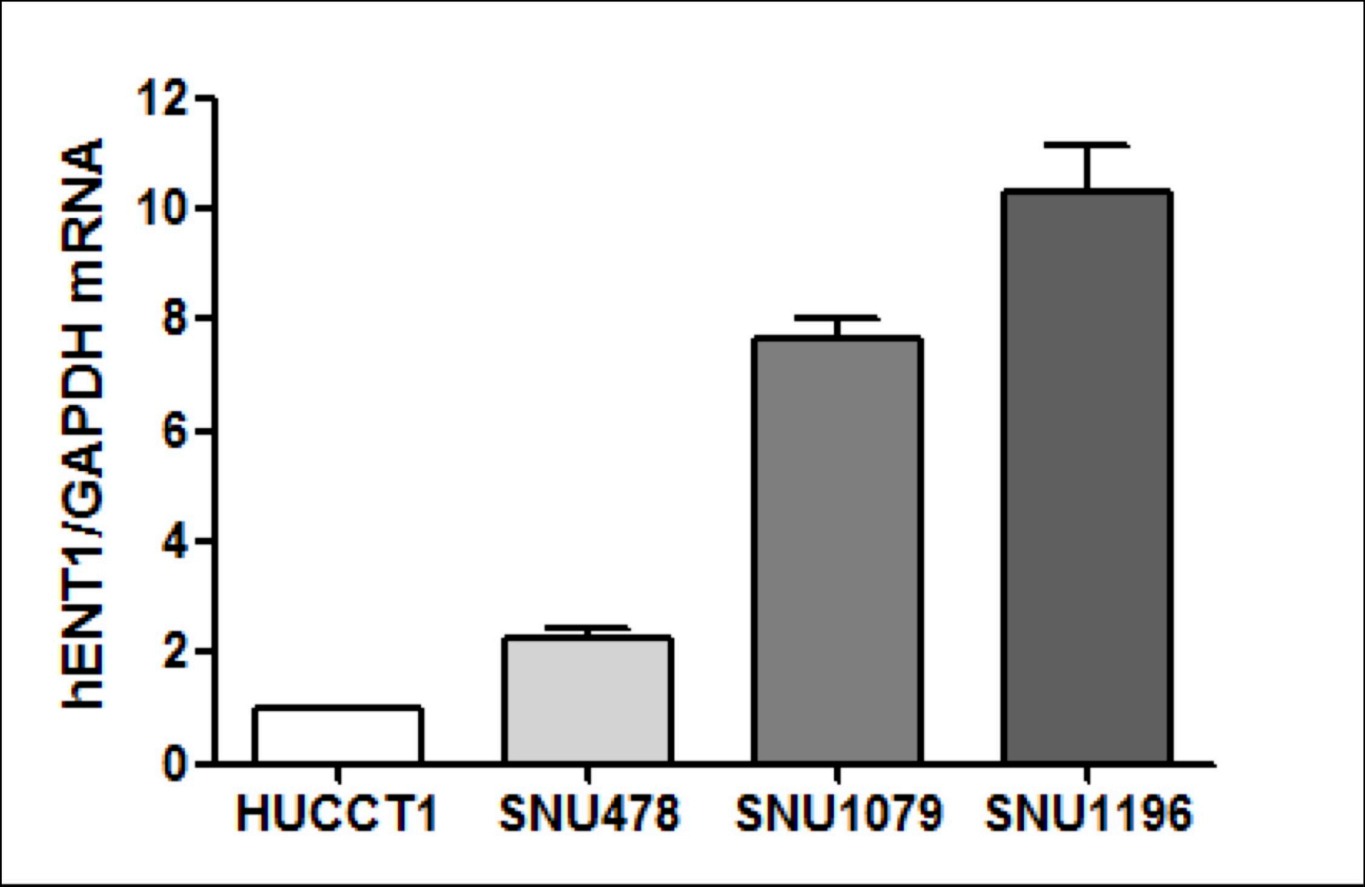
Expression of *hENT1* mRNA. Baseline levels of mRNA expression of *hENT1* were measured relative to those of HuCCT1 cells by quantitative RT-PCR. SNU1196 showed the highest mRNA expression of *hENT1*.

**Fig 2:**
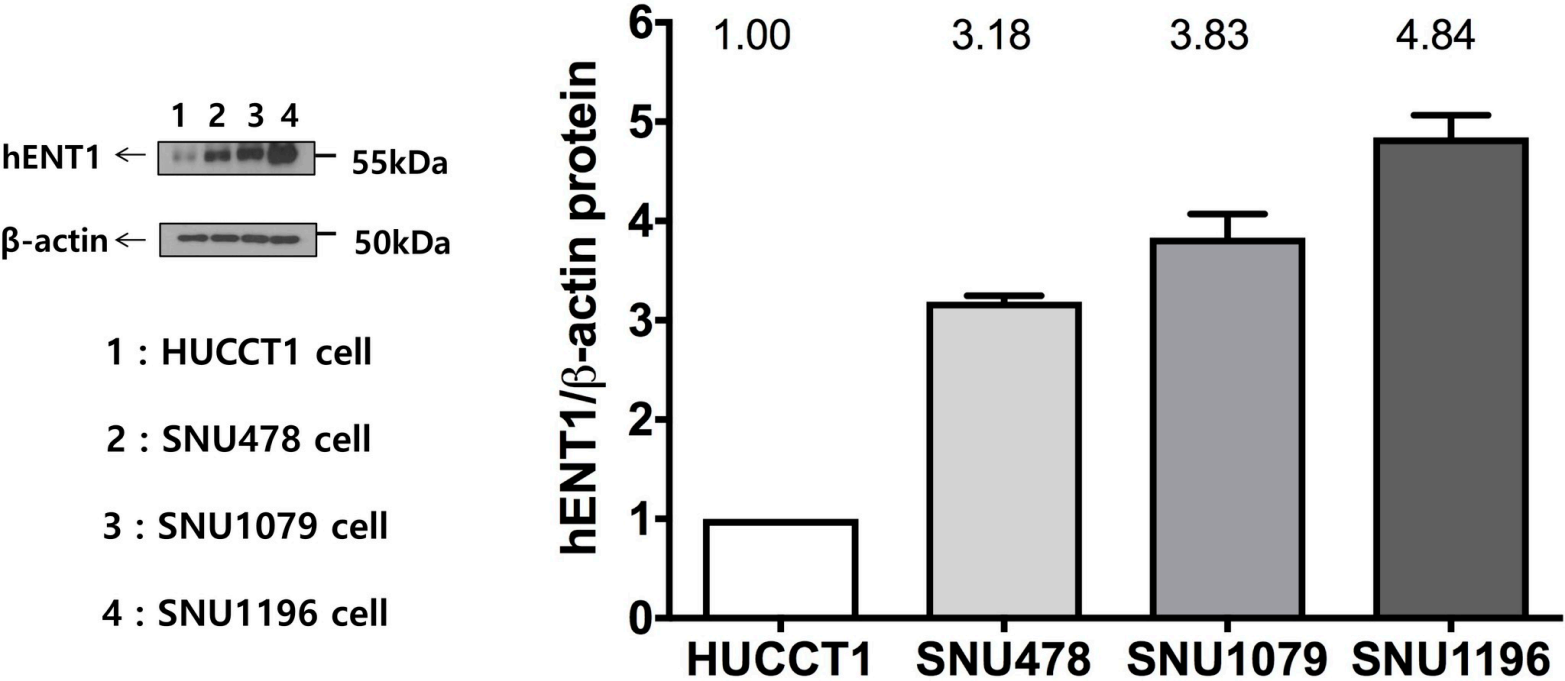
Expression of the hENT1 protein. Baseline levels of protein expression of hENT1 were measured relative to those of HuCCT1 cells by western blotting. SNU1196 showed the highest protein level of hENT1.

The baseline mRNA expression of *hENT1* in each cell line was measured in comparison to that in HuCCT1 cells by quantitative RT-PCR (Fig 1). Similarly, baseline levels of the hENT1 protein in each cell line were measured relative to HuCCT1 cells by western blotting (Fig 2). Among the four cell lines, SNU1196 showed the highest mRNA and protein levels of hENT1, whereas HuCCT1 had the lowest levels. The expression pattern of *hENT1* mRNA was similar to that of the hENT1 protein among the four cell lines.

### 2. The chemosensitivity assay

**Fig 3:**
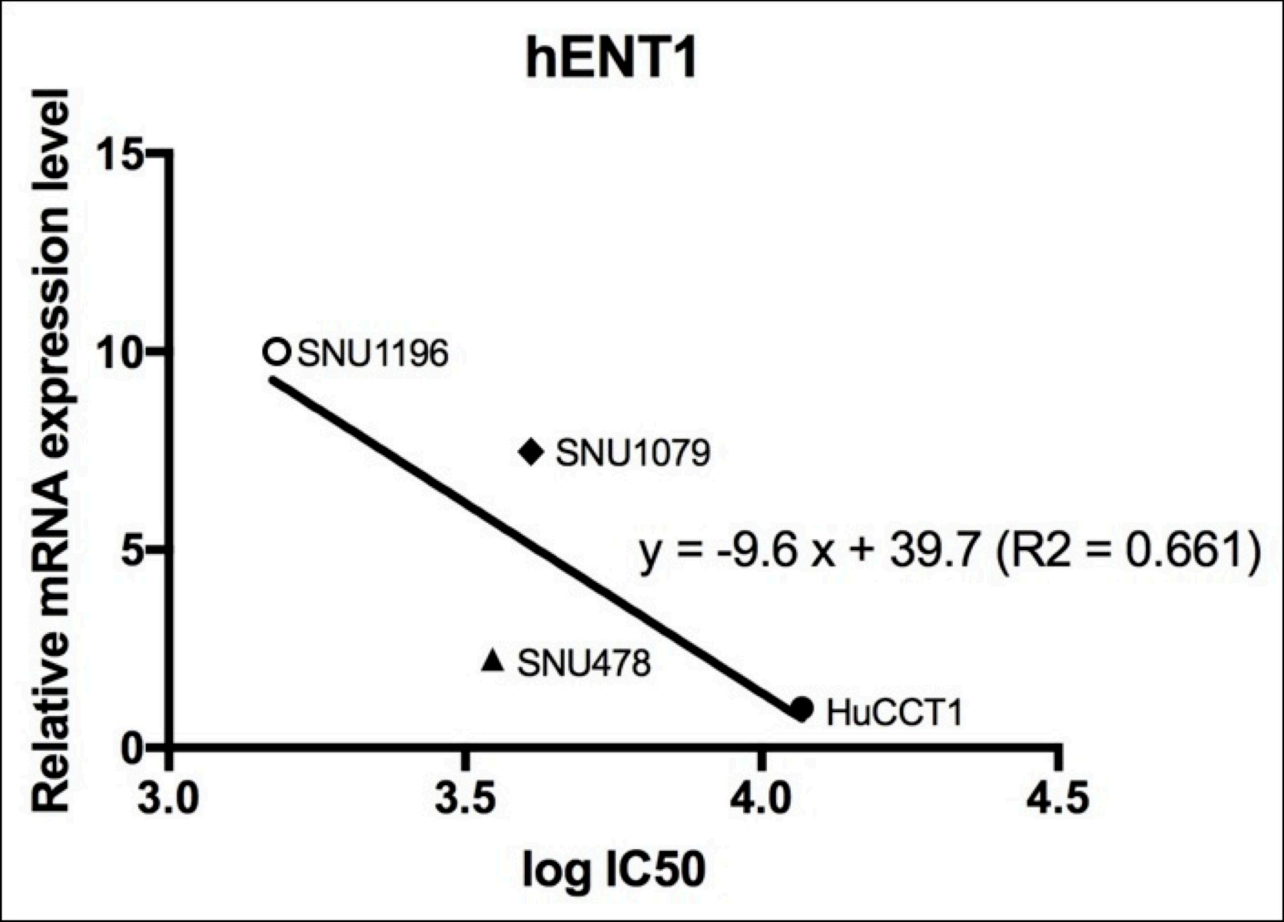
Correlation between gemcitabine chemosensitivity and basal expression of *hENT1* mRNA. Expression of *hENT1* mRNA showed a clear linear correlation with the log value of the half-maximal inhibitory concentration (IC_50_).

Among the four BTC cell lines, sensitivity to gemcitabine was measured as IC_50_. This parameter was the highest in HuCCT1 cells and the lowest in SNU1196 cells (Table 1 and S1 Fig). In the analysis of correlation between gemcitabine chemosensitivity and basal expression of *hENT1* mRNA, mRNA expression manifested a clear linear correlation with the log value of IC_50_ (Fig 3).

**Table 1:** Half-maximal inhibitory concentration (IC_50_) of gemcitabine in four biliary tract cancer (BTC) cell lines.

	HuCCT1	SNU478	SNU1079	SNU1196
**IC_50_ (nM) (mean ± SD)**	11682.5 **±** 632.3	3516.4 **±** 928.9	4093.5 **±** 202.3	1498.9 **±** 24.1

SD, standard deviation

### 3. Inhibition of cancer cell viability by hENT1 siRNA during gemcitabine treatment

**Fig 4:**
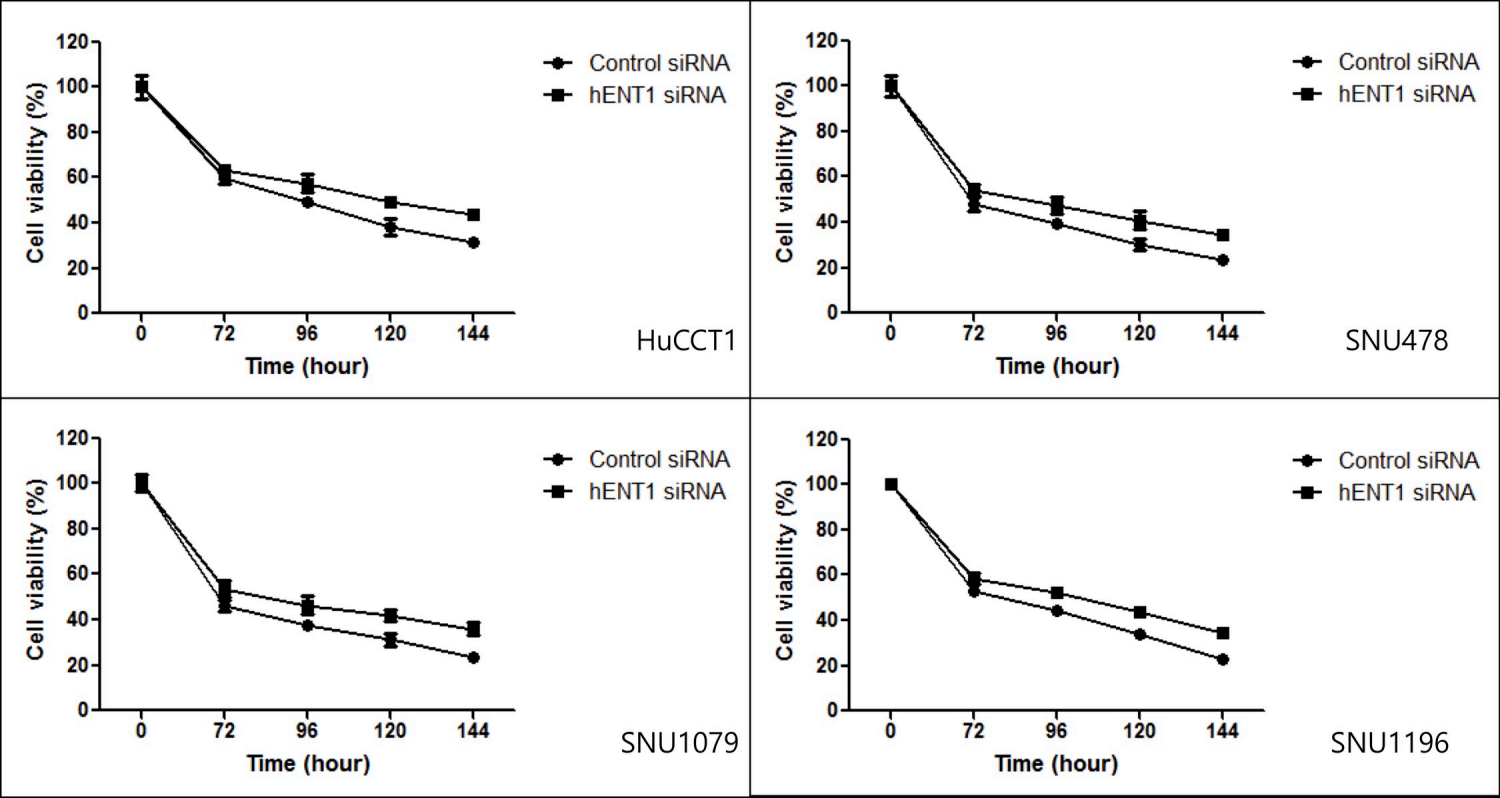
Effects of hENT1 siRNA on sensitivity of BTC cells to gemcitabine. Pretreatment with hENT1 siRNA was conducted to assess the effect of the hENT1 knockdown on gemcitabine sensitivity. During incubation with gemcitabine, in all cell lines, viability after hENT1 siRNA treatment was higher than that after control siRNA treatment.

**Fig 5:**
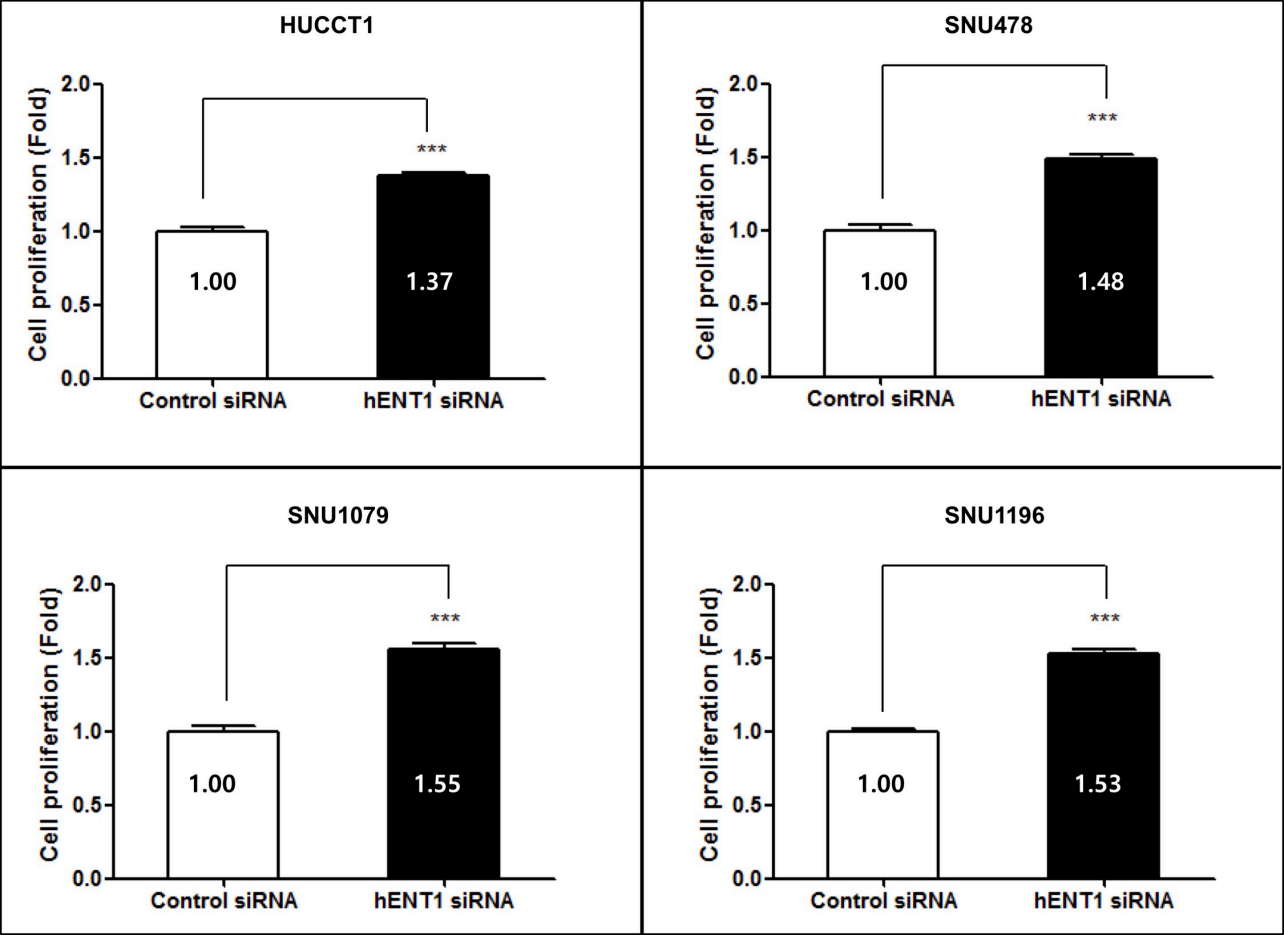
Effects of hENT1 siRNA on gemcitabine sensitivity. During incubation with gemcitabine, cell proliferation at 144 h after treatment with hENT1 siRNA was significantly higher than that after control siRNA treatment.

Pretreatment with hENT1 siRNA was conducted to assess the influence of hENT1 on the toxic effects of gemcitabine. In all BTC cell lines, viability after hENT1 siRNA treatment was higher than that after control siRNA treatment (Fig 4). Cancer cell proliferation at 144 h after treatment was significantly higher after hENT1 siRNA treatment than after control siRNA treatment (Fig 5). There was no difference in the inhibition of cell proliferation among the BTC cell lines.

### 4. Clinical outcomes

**Fig 6:**
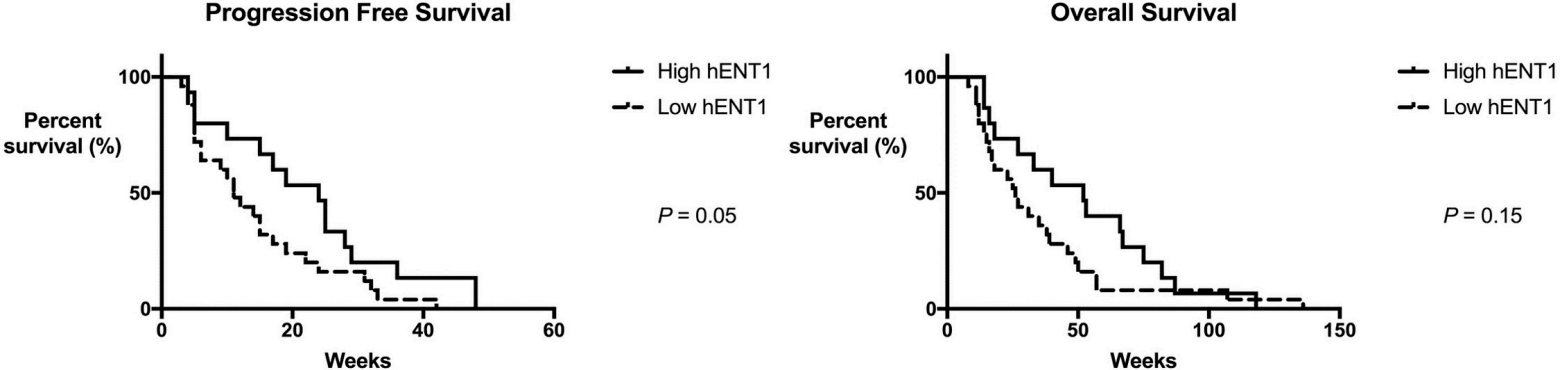
**(A) Median progression-free survival (PFS) of patients administered gemcitabine and cisplatin combination chemotherapy.** There was borderline significance of the difference in PFS between high- and low-hENT1 groups (24 and 11 weeks, P = 0.05). **(B) Median overall survival of patients who received gemcitabine and cisplatin combination chemotherapy.** There was no significant difference in overall survival between high- and low-hENT1 groups (52 and 26 weeks, P = 0.15).

According to hENT1 immunohistochemical staining grades, there were 15 patients with grade 2+ in the tumor, nine with grade 1+, and 16 with grade 0. As a result, there were 15 high-hENT1 and 25 low-hENT1 patients (Table 2). There were 24 men and 16 women with a median age of 62.5 years (43–80 years). As for tumor location, 22 BTCs were diagnosed as intrahepatic, five as perihilar, 11 as extrahepatic cholangiocarcinoma, and two as gallbladder cancers without a significant difference in hENT1 expression among these types. Six patients underwent curative surgery, and five patients were treated with palliative surgery. Between initially unresectable and recurrent BTCs, there were no differences in progression-free survival (PFS; median 20.6 vs 12.9 weeks, P = 0.87) or overall survival (median 51.5 vs 27.0 weeks, P = 0.71). Besides, there were no significant differences in clinicopathological factors such as cellular differentiation, median chemotherapy cycle, and CA 19–9 levels between the two groups. Eleven patients were administered second-line chemotherapy with various regimens based on 5-fluorouracil after failure of the GP regimen. Nevertheless, there was no difference in the number of patients receiving second-line chemotherapy. Survival analysis was performed to compare the hENT1 groups. PFS was 24 and 11 weeks in the high- and low-hENT1 groups, respectively (P = 0.05, Fig 6A). The median overall survival was 52 and 26 weeks (P = 0.15, Fig 6B), respectively.

**Table 2:** Baseline characteristics of patients.

	High hENT1 (n = 15)	Low hENT1 (n = 25)	*P*
**Male (%)**	8 (53.3)	16 (64.0)	0.51
**Median age (range)**	61 (51–70)	63 (43–80)	0.61
**IC/PC/EC/GBC**	10/1/3/1	12/4/8/1	0.66
**Unresectable/Recurred patients**	12/3	22/3	0.65
**Cellular differentiation (Well/Moderate/Poorly/NA)**	0/6/3/6	1/17/3/4	0.54
**Initial operation (number of patients)**	Pancreatico-duodenectomy (1) Segmentectomy (2)	Pancreatico-duodenectomy (2) Extended left lobectomy (1)	
**Median GP cycle (range)**	6 (2–12)	3 (1–18)	0.10
**Counts of patients with second line chemotherapy (number of patients with regimen)**	5 (iFAM 1, FOLFIRI 1, XP 3)	6 (iFAM 2, XP 4)	0.17
**Median Pre-treatment CA 19–9 (range)**	167.7 (6–20000)	740.0 (0.6–20000)	0.82
**Pre-treatment CA 19–9 > 37 (%)**	11 (73.3)	20 (80.0)	> 0.99

hENT1, human equilibrative nucleoside transporter 1; IC, intrahepatic cholangiocarcinoma; PC, perihilar cholangiocarcinoma; EC, extrahepatic cholangiocarcinoma; GBC, gallbladder carcinoma; NA, not applicable; GP, gemcitabine and cisplatin; iFAM, infusional 5-fluorouracil, doxorubicin, and mitomycin C; FOLFIRI, irinotecan with 5-fluorouracil and folinic acid; XP, capecitabine and cisplatin; CA 19–9, carbohydrate antigen 19–9

## Discussion

Gemcitabine is a chemotherapeutic agent widely used for many malignant tumors [8, 10, 11, 13]. Because of its role as an intracellular transporter of gemcitabine, hENT1 has been examined as a candidate cancer biomarker, particularly in pancreatic cancer [8, 9, 15–19]. Compared to pancreatic cancer, few studies on BTC have examined the combination of hENT1 and gemcitabine because of the low incidence of BTC. Moreover, the results have been inconsistent because various investigators employed either palliative [12, 20] or adjuvant [14] settings with different regimens. Therefore, we focused on patients with advanced BTC receiving the current first-line GP regimen as palliative chemotherapy.

In the gemcitabine metabolic pathway, several key proteins may be regarded as predictive biomarkers of a response to gemcitabine chemotherapy. Among them, hENT1 was evaluated in this study because its clinical value has been widely examined in pancreatic cancer research [8, 9, 16, 19]. Among the four BTC cell lines, we detected a linear correlation between the basal expression of *hENT1* mRNA and sensitivity to gemcitabine. Unexpectedly, when the expression of hENT1 was knocked down, chemoresistance was found to be similar among the four BTC cell lines, regardless of their different basal expression levels of *hENT1* mRNA. This result suggests that hENT1 acts as a threshold in the transport of gemcitabine; however, further studies are necessary to confirm this finding.

In addition to the *in vitro* results, the correlates of intratumoral hENT1 expression in this study point to its usefulness as a predictive biomarker. Although the clinical value of hENT1 in the transport of gemcitabine in many cancers has been demonstrated by immunohistochemical staining elsewhere [9–11, 18, 19, 21, 22], there is no standardized method for discrimination among clinical outcomes. Therefore, we adopted an hENT1 immunostaining method widely accepted in pancreatic cancer research [15] because of the similar clinical and genetic features of these diseases. Then, we set a cutoff value to discriminate between high and low expression of hENT1 in relation to clinical outcomes. As a result, hENT1 was found to be associated only with PFS, in agreement with other studies on BTC [12–14], and borderline significance of the difference in PFS (24 and 11 weeks, P = 0.05) was observed between the high- and low-hENT1 groups, although there was no significant difference in overall survival (52 and 26 weeks, P = 0.15). These data are consistent with the results of another study, which revealed better time to progression in high-hENT1 patients without clinical significance of overall survival in 31 patients [12]. Despite low statistical power due to the small number of patients here, intratumoral hENT1 expression in this study was found to be a predictive marker independent from other clinicopathological factors [14].

This study has several strengths. First, we conducted both cell line experiments and clinical analysis. Owing to the limited number of studies on BTC cell lines, our results are important because we demonstrated that the experimental and clinical results were consistent. Second, all the patients in this study received the same chemotherapy regimen (gemcitabine 1000 mg/m^2^ and cisplatin 25 mg/m^2^) that was used in the ABC-2 trial [3]. Finally, we found a direct correlation between hENT1 expression and sensitivity of BTC to gemcitabine, and demonstrated the suppressive effects of an hENT1 knockdown (via siRNA) on gemcitabine sensitivity of BTC cells.

This study has certain limitations. First, no *in vivo* experiments were conducted. Second, there is a possibility of a type II error in survival analysis because of the limited number of patients following the standard GP regimen. Small sample sizes have limited the conclusions of previous BTC studies as well.

Thus, our experimental results suggest that higher hENT1 expression is associated with a stronger toxic effect of gemcitabine on BTC cell lines. The clinical outcomes suggest that increased hENT1 expression in the BTC tumor is a potential biomarker predicting a better response to gemcitabine among patients with advanced BTC. As a result, it is possible to speculate on the possible utility of this marker for identifying the patients who would not benefit from gemcitabine-based chemotherapy. Further studies are necessary to determine the usefulness of hENT1 as a predictive marker and to develop effective therapeutic strategies against BTC.

## Supporting information

S1 Fig Cell viability after gemcitabine treatment in four biliary tract cancer (BTC) cell lines. (TIFF)

S1 Table Clinical dataset of enrolled patients. (XLSX)
