# Supplementary figures and images for: Human equilibrative nucleoside transporter 1 (hENT1) expression as a predictive biomarker for gemcitabine chemotherapy in biliary tract cancer
Source: PLoS One. 2018 Dec 17;13(12):e0209104. doi: 10.1371/journal.pone.0209104 (PMC6296552; doi:10.1371/journal.pone.0209104)

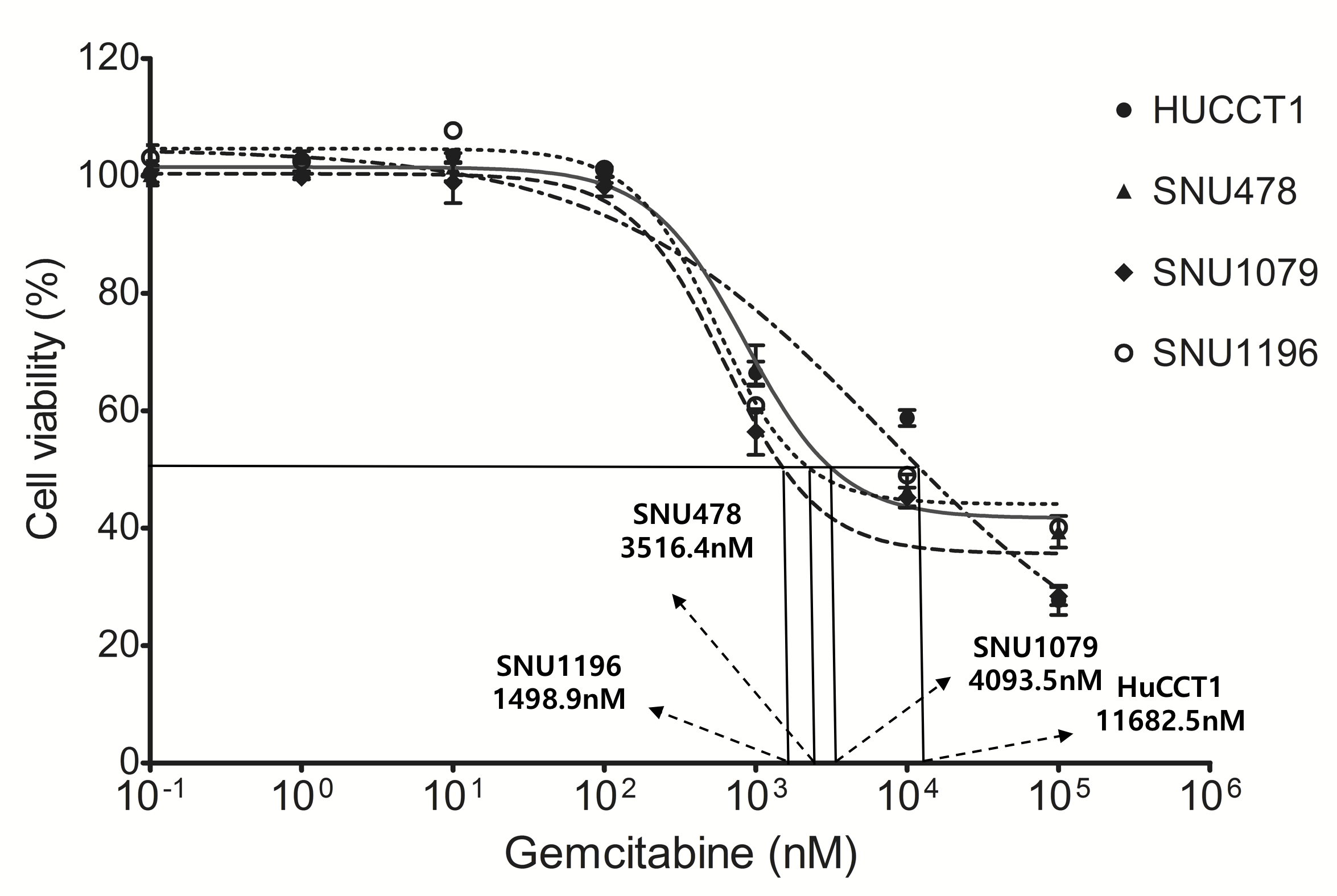

Supplement: S1 Fig — (TIFF) [file pone.0209104.s001.tiff]
